# Anxiety and depression in maintenance hemodialysis patients: prevalence and their effects on health-related quality of life

**DOI:** 10.1007/s11255-023-03556-7

**Published:** 2023-04-02

**Authors:** Eman Nagy, Samar Tharwat, Abdelrahman Mohamed Elsayed, Shimaa Abd El-Galeel Shabaka, Mohammed Kamal Nassar

**Affiliations:** 1 Mansoura Nephrology and Dialysis Unit, Department of Internal Medicine, Faculty of Medicine, Mansoura University, Mansoura, Egypt; 2 Rheumatology and Immunology Unit, Department of Internal Medicine, Faculty of Medicine, Mansoura University, Mansoura, Egypt; 3 Faculty of Medicine, Mansoura University, Mansoura, Egypt

**Keywords:** Anxiety, Depression, Hemodialysis, Quality of life, Mental disorders

## Abstract

**Purpose:**

The aims of the study are to explore the prevalence and risk factors of anxiety and depression in hemodialysis (HD) patients and to study their relationship with quality of life (QOL).

**Methods:**

This cross-sectional study involved 298 HD patients. Sociodemographic, clinical, and laboratory data of the patients were obtained from their records. Anxiety and depression were assessed by utilizing Hospital Anxiety and Depression Scale (HADS). In addition, QOL of the patients were evaluated by fulfilling the Kidney Disease Quality of Life-36.

**Results:**

This study included 298 HD patients (male 59.1%) with a median age of 49 years. Abnormal and borderline cases of anxiety were recognized in 49.6%, 26.2% of the patients, respectively, while depression cases and borderline cases were identified in 55 and 28.2% of the patients, respectively. Percentages of females (41 and 48% vs 26.4%, respectively), and patients who were not working (92.3 and 93.9% vs 72.2%, respectively) increased significantly in borderline and abnormal anxiety groups. Patients who did not work, led an inactive lifestyle, and smoked had considerably greater percentages in the borderline and abnormal HADS-depression categories than normal patients. Abnormal cases of depression and anxiety had significantly longer duration of HD than other two groups. Abnormal and borderline cases of anxiety and depression had worse QOL components than the normal patients.

**Conclusion:**

Anxiety and depression are prevalent among HD patients in Egypt, and several sociodemographic and clinical risk factors are associated. In addition, these mental disorders are associated with poor QOL.

## Introduction

End stage kidney disease (ESKD) is a global public health problem since the patients must live on hemodialysis (HD) for the remainder of their lives unless they have a successful kidney transplant [1]. In comparison to the general population, HD patients had a higher mortality rate due to several factors that might negatively affect these patients' health [2]. Psychiatric disorders are common in patients with chronic illnesses including ESKD and HD patients. Anxiety and depression are two of these conditions [3–6], with estimates ranging from 20 to 45% [7–9] and 25 to 50% [3, 5, 8, 10] in HD patients, respectively.

These diseases can lead to poor dialysis adherence and disruptive attitudes, such as missed HD treatments and shorter HD session duration [11–13]. Furthermore, these mental problems are linked to an increase in suicide, hospitalization, and death [12, 14]. Moreover, psychological, physical, and lifestyle problems have been shown to have an impact on HD patients' health-related quality of life (HRQOL) [15, 16]. Depression has been linked to demographic, socioeconomic, and clinical risk variables such as younger age, female gender, lower educational level, unemployment, hypertension, smoking status, and diabetes in HD patients [17, 18].

The Hospital Anxiety and Depression Scale (HADS) questionnaire is used to assess anxiety and depression in patients with various medical disorders. The HADS self-assessment tool is designed for early detection of potential mental disease that may improve with therapy [19].

To best of our knowledge, there are few studies which addressed prevalence and determinants of anxiety and depression in HD patients in Egypt. Thus, the aims of the current study were to explore the prevalence and risk factors of these psychiatric disorders in Egyptian HD patients and to discover the relationship between these disorders and HRQOL in those patients.

## Patients and methods

This multi-center cross-sectional study comprised 298 HD patients recruited from three different HD units in Egypt's Dakahlia and Gharbia governorates between January and May 2021. Adult patients who have been on HD for more than 3 months met the inclusion criteria. Patients with cognitive dysfunction, terminal cancer, severe organ dysfunction, hearing or visual impairments were not eligible for the trial. The sample size was selected as a convenience sample; all patients who met the inclusion criteria were invited to participate in the study, unless they were excluded by any of the exclusion criteria or declined to participate. Informed written consent was obtained from all participants prior to their enrollment in the study. The study was approved by the Mansoura Faulty of Medicine Institutional Research Board (Approval number R.22.07.1767).

Patients' sociodemographic data, such as age, gender, marital status, residence, level of education, job position, socioeconomic status (SES), and smoking behaviors, were collected. In addition, clinical characteristics such as hemodialysis duration, the existence of any comorbidities, and medication history were documented.

### Blood sampling and laboratory tests

Just before starting the first HD session of the week, blood samples were taken from the arteriovenous fistula. An automated analyzer was used to perform routine laboratory tests on the same day of blood sampling.

### Hospital anxiety and depression scale (HADS)

The patients completed an Arabic-language version of the HADS questionnaire. The HADS questionnaire is a tool that uses two separate subscales to assess anxiety and depression. The HADS has proven to be a valid instrument for use in general population [20], patients with different diseases [21–23] and patients with kidney disease [24, 25]. All the questions are graded on a four-point Likert scale (from 0 to 3). Patients with a score of 7 or less are deemed normal for depression and anxiety, those with a score of 8–10 are considered borderline for depression and anxiety (borderline cases), and those with a score of 11–21 are considered abnormal for depression and anxiety (cases) [26].

### The Kidney Disease Quality of Life-36 (KDQOL-36™)

KDQOL-36 was used to assess HRQOL of the studied patients. The original version included the Medical Outcomes Study 36 as a generic chronic illness core, as well as items specific to kidney disease patients. It consisted of 36 questions, with questions 1–12, 13–16, 17–28, and 29–36 yielding the mental health composite (MHC), physical health composite (PHC), burden of renal disease, symptom/problem list, and effect of kidney disease components of QOL, respectively. The average values for these five KDQOL-36 components range from 0 to 100, with higher scores suggesting better HRQOL. [27].

### Statistical analysis

Parametric and non-parametric continuous data were expressed as mean ± SD and median (minimum–maximum), respectively. Categorical data were expressed as number (percentage). Kolmogorov–Smirnov test was used to test for normality. Chi-square test was used to compare categorical variables. Analysis of Variance (ANOVA) test was used to compare parametric variables, while Kruskal–Wallis one-way analysis of variance was used to compare non-parametric variables between 3 subgroups of anxiety and depression. Univariate and multivariate linear regression analysis were used to discover the most significant associated factors of anxiety, depression and HRQOL in this study. P value less than 0.05 was considered to be significant. The tests were performed using SPSS 25 for personal computers.

## Results

In the current study, 298 HD patients were included, with a median age of 51 years and a male preponderance (59.1%). Table 1 shows the sociodemographic, clinical, therapeutic, and laboratory features of the individuals investigated. The bulk of the patients (71.8%) were married, and almost one-quarter were illiterate. Less than half of the patients (47%) were from low-income families. HD lasted an average of 5 years. The majority of patients (61.4%) had hypertension, 14.4% had diabetes, and 13.4% had no comorbidities. Calcium and iron supplements were given to the great majority of patients (78.9% and 73.2%, respectively). Patients' hemoglobin levels averaged 10.47 gm/dl, while their serum calcium, phosphorus, and albumin levels were all within normal limits. TSAT was lower than expected, although serum ferritin and iPTH were higher (Table 1).

**Table 1:** Sociodemographic, clinical, therapeutic and laboratory data of the studied patients (*n* = 298)

Variable	All (*n* = 298)
**Sociodemographic data**	
Age (years)	51(19–90)
Gender	
Male	176(59.1%)
Female	122(40.9%)
Married	214(71.8%)
Live alone	51(17.1%)
Residence	
Urban	140(46.9%)
Rural	158(53.1%)
Educational level	
Non-educated	73(24.5%)
Low school	33(11.1%)
Middle school	42(14.1%)
High school	109(36.6%)
College degree	37(12.4%)
Post-graduate	4(1.3%)
Occupational status	
Working	35(11.7%)
Not working	263(88.3%)
Active lifestyle	53(17.8%)
Socioeconomic status	
Low	140(47%)
Average	153(51.3%)
High	5(1.7%)
Smoking	
Non-smoker	255(85.6%)
Smoker	43(14.4%)
**Clinical data**	
Time since starting HD (years)	5(0.1–30)
Associated comorbidities	
No comorbidities	40(13.4%)
Diabetes	43(14.4%)
Hypertension	183(61.4%)
Psychiatric	9(3%)
COPD	4(1.3%)
IHD	16(5.4%)
**Therapeutic data**	
No medications	2(0.7%)
Erythropoietin stimulating agents	155(52%)
Calcium supplements	235(78.9%)
Iron supplements	218(73.2%)
Antihypertensives	188(63.1%)
Antidiabetics	59(19.8%)
**Laboratory data**	
Blood hemoglobin (gm/dl)	10.47 ± 1.3
Serum albumin (gm/dl)	4(3–4.5)
Serum calcium (mg/dl)	8.5(5.2–13)
Serum phosphorus (mg/dl)	4.6(1.9–10.6)
iPTH (pg/ml)	324.5(4–1991)
TSAT (%)	21(5–59)
Serum ferritin (ng/ml)	281.3(12.6–1375.9)

*HD* hemodialysis, *COPD* chronic obstructive pulmonary disease, *IHD* ischemic heart disease, *iPTH* intact parathyroid hormone, *TSAT* transferrin saturation

The data represented as median (min–max), mean ± SD or number (percentage)

Regarding HADS-anxiety, the median score was 11, and nearly half of the patients were anxious (49.6%), 26.2% of patients were borderline anxious, and 24.2% were normal. The median HADS-depression score was 10, with normal, borderline, and abnormal cases accounting for 16.8, 28.2, and 55% of the patients, respectively. Table 2 also included data on the patients' HRQOL.

**Table 2:** HADS score and HRQOL in the studied patients

Variable	The patients (*n* = 298)
**HADS-Anxiety:** median (min–max)	11(0–21)
Normal	72(24.2%)
Borderline	78(26.2%)
Abnormal	148(49.6%)
**HADS-Depression:** median (min–max)	10(0–20)
Normal	50(16.8%)
Borderline	84(28.2%)
Abnormal	164(55%)
**HRQOL domains**	
Symptom/problem list	70.83(0–100)
Effect of kidney disease	65.63(0–100)
Burden of kidney disease	15.62(0–100)
Physical health composite	32.64(16.2–57.90)
Mental health composite	36.59(12.8–60.7)

*HADS* hospital anxiety and depression scale, *HRQOL* health-related quality of life, *MHD* maintenance hemodialysis

The data presented as number (percentage) or median (min–max)

As regard HADS-anxiety subgroups, female percentages increased significantly in borderline and abnormal groups (41 and 48% vs 26.4%, respectively). Patients who were not working (92.3 and 93.9% vs 72.2%, respectively) and had an inactive lifestyle (87.2 and 88.5% vs 63.9%, respectively) were considerably more borderline and abnormal than normal patients. Smokers were substantially more common in the borderline and abnormal groups than in the normal group (20.5 and 16.9% vs 2.8%). The duration of HD in borderline and abnormal cases was much longer than in normal people (median was 6 and 6 vs 3 years). However, there were no statistically significant variations in age, marital status, living alone, residence, educational level, or socioeconomic status amongst the groupings. Serum phosphorus levels in normal people were substantially higher than in borderline and abnormal patients (5.1 vs 4.5 and 4.4 mg/dl, respectively). The remaining laboratory data, however, showed no significant variance (Table 3).

**Table 3:** Comparison between anxiety and depression subgroups regarding socio-demographic, medical and laboratory data

	HADS-anxiety				HADS-depression			
	Normal (*n* = 72)	Borderline (*n* = 78)	Abnormal (*n* = 148)	P	Normal (*n* = 50)	Borderline (*n* = 84)	Abnormal (*n* = 164)	*P*
**Sociodemographic data**								
Age	51(19–77)	49(27–83)	52(23–90)	0.975	45(21–71)	54(12–81)	48(7–90)	0.164
Gender								
Male	53(73.6%)	46(59%)	77(52%)	**0.009**	35(70%)	46(54.8%)	95(57.9%)	0.202
Female	19(26.4%)	32(41%)	71(48%)		15(30%)	38(45.2)	69(42.1%)	
Married	58(80.5%)	52(66.7%)	104(70.3%)	0.252	39(78%)	61(72.6%)	114(69.5%)	0.619
Live alone	11(15.2%)	13(16.6%)	27(18.2%)	0.857	5(10%)	14(16.7%)	32(19.5%)	0.358
Residence								
Urban	36(50%)	38(48.7%)	66(44.6%)	0.845	24(48%)	37(44%)	79(48.2%)	0.829
Rural	36(50%)	40(51.3%)	82(55.4%)		26(52%)	47(56%)	85(51.8%)	
Education								
Non-educated	14(19.4%)	16(20.5%)	43(29.1%)	0.194	7(14%)	25(29.8%)	41(25%)	0.128
Low school	7(9.7%)	6(7.7%)	20(13.5%)		3(6%)	12(14.3%)	18(11%)	
Middle school	11(15.3%)	9(11.5%)	22(14.9%)		8(16%)	6(7.1%)	28(17.1%)	
High school	24(33.3%)	35(44.9%)	50(33.8%)		21(42%)	27(32.1%)	61(37.2%)	
College degree	14(19.4%)	11(14.1%)	12(8.1%)		10(20%)	13(15.5%)	14(8.5%)	
Post-graduate	2(2.8%)	1(1.3%)	1(0.7%)		1(2%)	1(1.2%)	2(1.2%)	
Occupational status								
Working	20(27.8%)	6(7.7%)	9(6.1%)	** < 0.001**	12(24%)	5(6%)	18(11%)	**0.007**
Not working	52(72.2%)	72(92.3%)	139(93.9%)		38(76%)	79(94%)	146(89%)	
Active lifestyle	26(36.1%)	10(12.8%)	17(11.5%)	** < 0.001**	26(52%)	9(10.7%)	18(11%)	** < 0.001**
Socioeconomic status								
Low	36(50%)	38(48.7%)	66(44.6%)	0.636	24(48%)	42(50%)	74(45.1%)	0.682
Average	34(47.2%)	40(51.3%)	79(53.4%)		25(50%)	42(50%)	86(52.4%)	
High	2(2.8%)	0	3(2%)		1(2%)	0	4(2.4%)	
Smoking								
Non-smoker	70(97.2%)	62(79.5%)	123(83.1%)	**0.004**	49(98%)	73(86.9%)	133(81.1%)	**0.011**
Smoker	2(2.8%)	16(20.5%)	25(16.9%)		1(2%)	11(13.1%)	31(18.9%)	
**Clinical data **								
Time since starting HD	3(0.25–21)	6(0.5–24)	6(0.1–30)	**0.001**	3.5(0.25–25)	5(0.5–21)	6(0.1–30)	**0.013**
Associated comorbidities								
No comorbidities	10(13.9%)	8(10.3%)	22(14.9%)	0.621	5(10%)	14(16.7%)	21(12.8%)	0.517
Diabetes	12(16.7%)	13(16.7%)	18(12.1%)	0.612	7(14%)	13(15.4%)	23(14%)	0.872
Hypertension	41(56.9%)	48(61.5)	94(63.5%)	0.643	32(64%)	56(66.7%)	95(57.9%)	0.375
Psychiatric	0	5(6.4%)	4(2.7%)	0.065	1(2%)	2(2.4%)	6(3.7%)	0.739
COPD	2(2.8%)	1(1.3%)	1(0.7%)	0.456	2(4%)	0	2(1.2%)	0.148
**Therapeutic data**								
No medications	0	1(1.3%)	1(0.7%)	1	0	0	2(1.2%)	0.693
Erythropoietin	40(55.6%)	33(42.3%)	82(55.4%)	0.136	29(58%)	42(50%)	84(51.2%)	0.639
Calcium supplements	48(66.7%)	69(88.5%)	118(79.7%)	**0.005**	29(58%)	74(88.1%)	132(80.5%)	** < 0.001**
Iron supplements	42(58.3%)	61(78.2%)	115(77.7%)	**0.005**	22(44%)	65(77.4%)	131(79.9%)	** < 0.001**
Antihypertensives	41(56.9%)	49(62.8%)	98(66.2%)	0.408	32(64%)	61(72.6%)	95(57.9%)	0.075
Antidiabetics	12(16.7%)	17(21.8%)	30(20.3%)	0.718	8(16%)	19(22.6%)	32(19.5%)	0.643
**Laboratory data**								
Blood hemoglobin (gm/dl)	10.67 ± 1.4	10.55 ± 1.2	10.35 ± 1.3	0.272	10.39 ± 1.2	10.24 ± 1.1	10.59 ± 1.4	0.187
Serum albumin (gm/dl)	4(3–4.4)	4(3.6–4.3)	3.8(3.1–4.5)	0.148	4(3–4.4)	4(3.1–4.4)	3.9(3.4–4.5)	0.315
Serum calcium (mg/dl)	8.25(5.8–9.7)	8.55(6.6–10.1)	8.5 (5.2–13)	0.394	8.3(6.2–9.7)	8.3(6–9.8)	8.5(5.2–13)	0.059
Serum phosphorus (mg/dl)	5.1(2.6–10.6)	4.5(2–10.2)	4.4(1.9–10.6)	** < 0.001**	5.1(2.1–10.2)	4.6(2.4–9.5)	4.4(1.9–10.6)	**0.034**
iPTH (pg/ml)	257(36.9–1559)	375(44.6–1991)	324.5(4–1978)	0.950	398.5(36.9–1991)	327.5(4.6–1149)	304.1(4–1978)	0.279
TSAT (%)	22(8–45)	21(5–39)	21(7–59)	0.575	22(5–45)	21(12–59)	21(7–39)	0.776
Serum ferritin (ng/ml)	364.3(13.5–797.3)	251(12.6–1375.9)	353.28(53.8–1139.7)	0.884	316.3(12.6–1375.9)	280(71.4–848.4)	282.6(13.5–1139.7)	0.783

The data represented as median (min–max), mean ± SD or number (percentage)

The bold *P* values mean that they were statistically significant

*HD* hemodialysis, *COPD* chronic obstructive pulmonary disease, *IHD* ischemic heart disease, *iPTH* intact parathyroid hormone, *TSAT* transferrin saturation

Patients who did not work, led an inactive lifestyle, and smoked had considerably greater percentages in the borderline and abnormal HADS-depression categories than normal patients. Age, gender, marital status, living alone, residence, educational level, and socioeconomic status, on the other hand, did not show statistically significant variations. The duration of HD in borderline and abnormal cases was much longer than in normal people (median was 5 and 6 vs 3.5 years). Serum phosphorus levels in normal people were significantly higher than in borderline and abnormal patients (5.1 vs 4.6 and 4.4 mg/dl, respectively). However, there was no significant variation in the remaining laboratory results (Table 3).

When the significant socioeconomic, clinical, and laboratory variables from the HADS-anxiety and depression subgroups were entered into a multiple linear regression analysis equation, the female gender and cigarette smokers were the most significant predictors of anxiety (Beta = − 0.270 and 0.134, *P* =  < 0.001 and 0.043, respectively). While inactive life style, female gender, and cigarette smokers were the most significant predictors of depression (Beta = − 0.227, − 0.169, and 0.135, *P* =  < 0.001, 0.009, and 0.041, respectively) Table 4.

**Table 4:** Univariate and multivariate linear regression analysis of HADS-anxiety and HADS-depression

	HADS-anxiety						HADS-depression					
	Univariate analysis		Multivariate analysis				Univariate analysis		Multivariate analysis			
	Beta	*P* value	Beta	*P* value	Confidence interval		Beta	*P* value	Beta	*P* value	Confidence interval	
					Lower bound	Higher bound					Lower bound	Higher bound
Female gender	− 0.174	**0.003**	− 0.270	** < 0.001**	− 3.084	− 1.112	− 0.119	**0.040**	− 0.169	**0.009**	− 2.037	− 0.301
Cigarette smoker	0.152	**0.009**	0.134	**0.043**	0.046	2.780	0.140	**0.016**	0.135	**0.041**	0.053	2.504
Inactive lifestyle	− 0.241	** < 0.001**	− 0.123	0.056	− 2.609	0.081	− 0.277	** < 0.001**	− 0.227	** < 0.001**	− 3.337	− 0.968
Duration of hemodialysis	0.135	**0.021**	0.086	0.189	− 0.033	0.164	0.161	**0.006**	0.105	0.107	− 0.016	0.161
Occupational status	0.084	0.146	− 0.070	0.272	− 2.633	0.744	0.029	0.619	0.049	0.439	− 0.919	2.109
Serum phosphorus	− 0.199	**0.001**	− 0.032	0.624	− 0.444	0.267	− 0.119	0.059	− 0.080	0.219	− 0.518	0.120

The bold *P* values mean that they were statistically significant

When the five KDQOL-36 components were examined across the HADS-anxiety and depression subgroups, the HRQOL component scores were considerably higher in normal patients compared to borderline and abnormal cases, indicating that normal patients have a better quality of life (Tables 5, 6). Univariate linear regression analysis were performed for all variables regarding HRQOL and showed that male gender, living alone, active lifestyle, anxiety, and depression were significant associates with HROL (Beta = 0.168, − 0.125, 0.173, − 0.459, and − 0.379, respectively, *P* = 0.004, 0.041, 0.003, < 0.001, and < 0.001, respectively). Entering these variables into multilinear regression analysis equation to identify the most significant associates of HRQOL resulted in exclusion of male gender, living alone and active lifestyle from being significant associates. Anxiety was the most significant associate of HRQOL (Beta = − 0.381, *P* =  < 0.001) followed by depression (Beta = − 0.142, *P* = 0.25) [Table 7].

**Table 5:** Health-related quality of life data of studied patients across HADS-anxiety subgroups

	Normal (*n* = 72)	Borderline (*n* = 78)	Abnormal (*n* = 148)	*P*
Symptom/problem list	81.25(25–100)	70.83(14.5–100)	64.58(0–100)	** < 0.001**
Effect of kidney disease	75(0–100)	64(12.5–100)	57.81(0–100)	** < 0.001**
Burden of kidney disease	25(0–87.5)	12.5(0–75)	12.5(0–75)	** < 0.001**
Physical health composite	35.71(16.2–56.5)	30.74(21.1–47.1)	32.13(18.6–57.9)	**0.007**
Mental health composite	45.37(24.6–60.7)	36.26(21.3–49.9)	35.04(12.8–49.5)	** < 0.001**

The data represented as median (min–max)

The bold *P* values mean that they were statistically significant

**Table 6:** Health-related quality of life data of studied patients across HADS-depression subgroups

	Normal (*n* = 50)	Borderline (*n* = 84)	Abnormal (*n* = 164)	*P*
Symptom/problem list	79.17(14.5–100)	66.67(4.1–100)	70.83(0–100)	**0.027**
Effect of kidney disease	75(0–100)	65.63(12.5–100)	59.38(0–100)	**0.001**
Burden of kidney disease	25(0–81.2)	12.5(0–81.2)	12.5(0–100)	** < 0.001**
Physical health composite	38.27(21.2–57.9)	32.48(18.6–51)	31.56(16.2–51.6)	** < 0.001**
Mental health composite	46.8(24.6–60.7)	36.72(21.3–58)	35.54(12.8–59.7)	** < 0.001**

The data represented as median (min–max)

The bold *P* values mean that they were statistically significant

**Table 7:** Univariate and multivariate linear regression analysis of total score of QOL

	Univariate analysis				Multivariate analysis			
	Beta	*P* value	Confidence interval		Beta	*P* value	Confidence interval	
			Lower bound	Higher bound			Lower bound	Higher bound
Gender (Male)	0.168	**0.004**	5.401	27.827	0.043	0.433	− 6.344	14.775
Live alone	− 0.125	**0.041**	− 31.518	− 0.657	− 0.078	0.144	− 23.511	3.455
Active lifestyle	0.173	**0.003**	7.795	36.959	0.083	0.134	− 3.652	27.156
Anxiety	− 0.495	** < 0.001**	− 7.415	− 4.931	− 0.381	** < 0.001**	− 6.203	− 3.181
Depression	-0.379	** < 0.001**	− 6.771	− 3.801	− 0.142	**0.025**	− 3.695	− 0.254

The bold *P* values mean that they were statistically significant

## Discussion

Anxiety and depression are common among HD patients [3, 5], and they have been linked to worse HRQOL [16]. Furthermore, certain recognized risk factors for these illnesses exist [17]. However, these disorders are understudied, with minimal research focusing on them in Egypt. As a result, the current study sought to determine the prevalence of anxiety and depression in Egyptian HD patients, as well as their determinants and links to HRQOL.

Anxiety was common among the patients in this study, with abnormal cases having a frequency of 49.6% and borderline cases having a frequency of 26.2%. Furthermore, depression was common, accounting for 55% of abnormal and 28.2% of borderline cases, respectively. The current study's prevalence of anxiety and depression is comparable to that of Kamel et al. [28] on 524 Egyptian HD patients. However, this finding is significantly higher than that obtained by Turkistani et al. [29], who evaluated 286 Saudi HD patients and discovered that 21.1 and 23.3% of patients, respectively, had elevated anxiety and depression scores. Furthermore, Mosleh et al. [30] showed that 19.7 and 24.6% of 122 HD patients, respectively, had abnormal anxiety and depression symptoms, which is much lower than our findings. Furthermore, the current prevalence is somewhat higher than that reported by Yoong et al. [8]. The aforementioned studies used HADS as a tool to assess anxiety and depression in dialysis patients. The difference in prevalence of these disorders between the studies might be due to different geographic areas and thence differences in socioeconomic statuses. Our prevalence is comparable to the prevalence mentioned by another Egyptian study but differs with other non-Egyptian studies.

In the current study, females reported significantly higher levels of anxiety than males. This finding is congruent with those of Gerogianni et al. [31], as well as Delgado-Domínguez et al. [32]. However, this finding contradicts the findings of Kamel et al. [28], Kao et al. [33] and Yoong et al. [8], who found no significant differences in anxiety levels between men and women in respective investigations. Several cognitive and physiological activities associated with anxiety have shown significant gender variations [34]. Estrogen and progesterone appear to have a significant impact on the activities of anxiety-related neurotransmitter systems and fear extinction [34], whereas testosterone has anxiolytic properties [35]. Females may be more prone to anxiety disorders than males due to variations in occupational level, socioeconomic situations, cultural restraints, and social responsibilities [36]. In addition, females in Arabic countries have more restrictions in lifestyle and more responsibilities than females. Thus, they might be more prone to anxiety disorders. Asher et al. concluded in their review that females report more social anxiety than men. They explained this conclusion by self-construal theory, and greater personal interaction and greater reduction in daily life satisfaction in women than men [37].

In the current study, smokers had significantly higher percentages of borderline and abnormal anxiety cases than nonsmokers. This conclusion contrasts the findings of Kamel et al., who discovered no changes in anxiety levels between smokers and nonsmokers [28]. Neurotransmitter systems, inflammation, oxidative and nitrosative stress, mitochondrial dysfunction, neurotrophins and neurogenesis, and epigenetic changes are thought to contribute to anxiety. Cigarette smoke components like as nicotine and free radicals have an impact on all of these pathways [38]. Another explanation of association of cigarette smoking with anxiety is that anxiety could lead to the urge to smoke.

Patients who were not working were more anxious than those who were, supporting the findings of Gerogianni et al. [31]. This finding, however, contradicts those of Kamel et al. [28], Kao et al. [33], Turkistani et al. [29], Delgado-Domínguez et al. [32], and Yoong et al. [8]. Non-work in working-age patients may be associated with a lack of financial means and, as a result, a loss of self-esteem. There may also be psychological concerns and anxiousness. In addition, it is possible that considerable anxiety makes it difficult for patients to work. Furthermore, time consuming in hemodialysis sessions could cause both anxiety and inability to work.

Patients with higher degrees of anxiety and depression (abnormal and borderline cases) had a significantly longer duration of HD in the current research. The findings of Kamel et al.'s [28] are consistent with ours. Our findings, on the other hand, contradict the findings of Gerogianni et al. [31], Delgado-Domínguez et al. [32], and Yoong et al. [8], who discovered no significant differences in HD duration among persons with varied levels of anxiety. Elkheir et al. [39] reported that individuals with HD who had it for less than a year were more depressed than those who had it for more than three years. HD is associated with negative concerns, such as fear of access and HD-related repercussions, which can create stress and worry in patients. Furthermore, frequent commuting and spending extended periods of time away from home may contribute to their stress levels.

The present study discovered that smokers had higher levels of depression than nonsmokers. This result differs from what Kamel et al. [28]. It is unclear if smoking causes depression or whether depression pushes people to start smoking. The relationship between the two is most likely troublesome. Nicotine causes the release of dopamine in the brain. Dopamine is responsible for happy feelings. It has been found repeatedly to be low among depressed persons, who may then turn to smoking to temporarily increase their dopamine levels. Smoking, on the other hand, causes the brain's natural dopamine-producing process to shut down, lowering the availability of dopamine in the brain over time and encouraging smokers to smoke more [40].

In the current study, non-workers had greater levels of depression than employees. This finding contradicts the findings of Kamel et al. [28], Donia et al. [41], Kao et al. [33], Delgado-Domínguez et al. [32] and Yoong et al. [8]. As mentioned before, unemployment may be related with financial difficulties, loss of self-esteem, and consequently depression.

Poor HRQOL was described in patients on HD [42–44]. In the current study, anxiety and depression were the most significant associated variables with HRQOL in multivariate linear regression analysis. Anxiety and depression have been associated to low HRQOL in several studies [15, 16, 41, 45, 46]. In the current study, both anxiety and depression had a negative influence on many parameters of HRQOL. Dialysis has a major and occasionally negative impact on patients' physical and mental health, impairing quality of life and causing psychological issues [46]. Low treatment adherence is thought to be one of the factors that contribute to the link between high levels of anxiety or depression and low quality of life [45]. The foregoing evidence suggests that HD patients who effectively avoid or treat anxiety and depression may improve significantly in terms of both HRQOL and regular physical activity [47].

The present results put an emphasis of evaluating mental disorders in HD patients in addition to searching for the underlying risk factors as a trial to address them because of the affliction of these disorders on HRQOL and health status of these patients. However, we recommend other studies to stratify the study cohort according to presence/absence of depression/anxiety.

Limitations of the current study included that anxiety and depression were assessed depending on self-report not on established clinical diagnosis. Additionally, convenience sampling is not representative of all population undergoing HD in Egypt and it could be the reason for the different prevalence of the disease from other studies. In addition, activity of lifestyle was collected from the patients’ own words so it might be inaccurate. Furthermore, the limited range of collected variables that might have impacts on the multivariable linear regression model of both anxiety and depression. Moreover, selection bias was one of the study limitations due to exclusion of patients who did not want to participate in the study which could be due to having some degree of anxiety/depression.

## Conclusion

Anxiety and depression are prevalent among Egyptian HD patients, and many sociodemographic and clinical risk factors, such as female gender, sedentary lifestyle, smoking, and longer HD duration, are associated with them. In addition, these mental disorders are associated with poor HRQOL in those patients.

## Data Availability

The dataset generated and analysed during the current study are available from the corresponding author on reasonable request.
